# Automating production of de-identified linkable data

**DOI:** 10.23889/ijpds.v9i5.2840

**Published:** 2024-09-18

**Authors:** Madalina Iova, Rachel Huck

**Affiliations:** 1 Office for National Statistics

## Objective

An innovative large-scale automated method has been developed to produce de-identified linkable data. The objective is to create a wide pool of ready-to-use data to enable faster and wider collaborative analysis for the public good.

## Approach

A configurable automated pipeline prepares data for onward linkage at location, person, business and classification level through:

Big data profiling pre- and post-processing, for overview of variables and characteristics
Flagging potentially sensitive/identifiable variables
Generalisable linkage methods for large-scale data, to enable the addition of unique IDs for onward linkage of de-identified data
De-identification, hashing and redaction mechanisms, to remove and/or obscure sensitive/identifiable variables
Automated production of metadata, capturing linkage quality and transformations across the data journey
Quality assurance checks, including measure of linkage quality, assurance of variable derivations and redactions, and consistency checks on remaining data.

## Results

The pipeline enables a configurable automated approach to producing de-identified, linkable, ready-to-use data in a traceable and fully documented manner. To achieve this we have: overcome scalability issues in working with big data; implemented automation at various levels; and enabled standardisation across data types and data structures to deliver a consistent recognisable final product.

## Conclusions and Implications

Building an automated pipeline to enable onward linkage of de-identified administrative data was a complex process that has resulted in positive change around how our organisation operates and distributes data. This represents an important step towards future integration of linkage within the platform and the basis of future innovation in the area.

